# A Surfeit

**Published:** 1877-07

**Authors:** 


### A SURFEIT

In man is called founder in a horse, and is over-eating, eating more than the stomach can possibly convert into healthful blood. Wise men, and careful men will sometimes inadvertently eat too much, known by a feeling of fulness, of unrest, of a discomfort which pervades the whole man. Under such circumstances, we want to do something for relief; some eat a pickle, others swallow a little vinegar, a large number drink brandy. We have swallowed too much, the system is oppressed, and nature rebels, instinct comes to the rescue, and takes away all appetite, to prevent our adding to the burden by a morsel or a drop. The very safest, surest, and least hurtful remedy, is to walk briskly in the open air, rain or shine, sun, hail, or hurricane, until there is a very slight moisture on the skin, then regulate the gait, so as to keep the perspiration at that point, until entire relief is afforded, indicated by a general abatement of the discomfort; but as a violence has been offered to the stomach, and it has been wearied with the extra burden imposed upon it, the next regular meal should be omitted altogether. Such a course will prevent many a sick hour, many a cramp, colic, many a fatal diarrhœa.

